# Molecular interactions in short-chain perfluoroalkyl carboxylic acids and aqueous solutions

**DOI:** 10.1098/rsta.2022.0333

**Published:** 2023-10-30

**Authors:** Chris J. Benmore, Yuqin Wang, Seth B. Darling, Junhong Chen

**Affiliations:** ^1^ X-Ray Science Division, Advanced Photon Source, Argonne National Laboratory, Lemont,IL 60439, USA; ^2^ Chemical Sciences and Engineering Division, Physical Sciences and Engineering Directorate, Argonne National Laboratory, Lemont,IL 60439, USA; ^3^ Consortium for Advanced Science and Engineering, University of Chicago, Chicago,IL 60637, USA; ^4^ Pritzker School of Molecular Engineering, University of Chicago, Chicago,IL 60637, USA

**Keywords:** liquid structure, PFAS, X-ray diffraction, pair distribution function

## Abstract

The presence of short-chain per- and polyfluoroalkyl substances in water poses a major health and environmental challenge. Here, we have performed high-energy small- and wide-angle X-ray scattering measurements on CF_3_[CF_2_]*_n_*COOH (where *n* = 1, 2, 3 represents the chain length) and their aqueous solutions at 10% mole concentrations to characterize their molecular interactions at the atomic and nanometer length scales. The experimental wide-angle structure factors have been modelled using Empirical Potential Structural Refinement. The oxygen–oxygen partial X-ray pair distribution functions show that the coordination number between the hydroxyl oxygen on the acid and surrounding oxygen water molecules increases significantly with acid chain length, rising from 3.2 for *n* = 1 to 4.1 for *n* = 3. The small-angle scattering is dominated by a sharp, high-intensity peak at *Q*_1_ ∼ 0.2 Å^−1^ and a smaller peak at *Q*_2 _= 1.2 Å^−1^ for *n* = 3, both of which decrease with decreasing chain length. The *Q*_2_ peak is attributed to groups of adjacent non-bonded acid molecules, and *Q*_1_ has contributions from both correlations between acid molecules and water–water interactions. In all cases, the models show nanoscale aggregation occurs in the form of denser channels of winding hydrogen-bonded chains, approximately 20 water molecules in length, surrounding clusters of acid molecules.

This article is part of the theme issue 'Exploring the length scales, timescales and chemistry of challenging materials (Part 2)'.

## 1. Introduction

**Figure 1.**
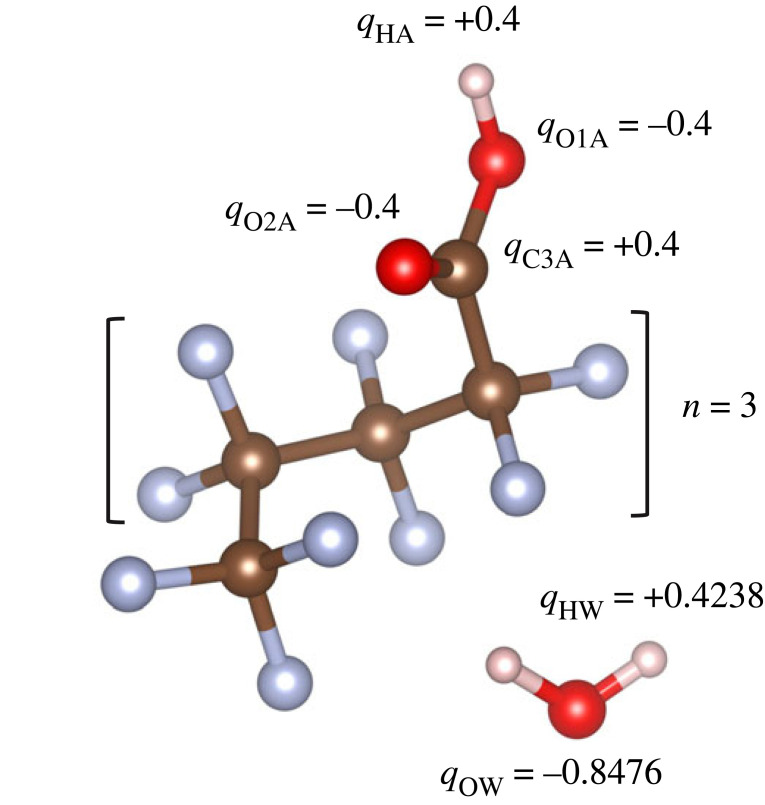
Structure of perfluoroalkyl carboxylic acid and water molecules used in the EPSR simulation including the partial charges and atom labels. Label O1A represents a hydroxyl oxygen and O2A a carbonyl oxygen. Acids with chain lengths of *n* = 1, 2 and 3 were studied.

Water contamination by per- and polyfluoroalkyl substances (PFAS) has become an increasingly severe environmental and health crisis. Exposure to PFAS chemicals can lead to adverse human health effects, since they are persistent in the environment due to the strong bonding between carbon and fluorine [1–5]. Moreover, since they can accumulate over time, the U.S. Environmental Protection Agency (EPA) has established and recently updated, health advisory levels to provide Americans, including the most vulnerable populations, with a margin of protection from a lifetime of exposure to PFAS [6]. It is estimated that over 200 million people likely receive water with a PFAS concentration at or above 1 ng l^−1^ [7]. Structurally, PFAS molecules each possess a hydrophobic ‘tail’ and a hydrophilic ‘head’, figure 1. Although long-chain PFAS are most commonly detected in the aquatic environment, their use has been limited by recent regulations, and more attention is being paid to the rise in alternative short-chain compounds. Short-chain PFAS have a high mobility in water bodies, and their removal by adsorption is more challenging [8,9]. The shorter chain length results in less hydrophobic interactions compared with long-chain PFAS, which is the dominant mechanism of capture making the remediation of short-chain PFAS much harder. It is therefore important to quantify both the hydrophobic and hydrophilic interactions of PFAS with water as a function of chain length.

To investigate these interactions associated with fluoroalkyl chains and water at the atomic scale, we have studied the structure of pentafluoropropionic acid, heptafluorobutyric acid, perfluoropentanoic acid and their aqueous solutions at 10% mole concentrations. Rat oral acute toxicity data from the EPA centre for computational toxicology and exposure database indicates that these short-chain fluorinated acids have lethal concentrations at approximately 0.1 g l^−1^ [10]. However, diffraction experiments at even these concentrations are untenable due to the weak signal. Nonetheless, the high solubility of short fluoroalkyl acid chains allows us to maximize the X-ray signal between acid and water and investigate the interactions with water. The characterization of the number and strength of hydrogen bonds, and hydrophobic interactions between short-chain PFAS and water molecules at high concentrations provide insights relevant to the environmental challenge of PFAS. It is anticipated that the details of the molecular interactions from such fundamental studies will provide stringent benchmarks for the development of interatomic potential parameters used in computer simulations that can be employed at much lower contaminant concentrations.

Diffraction studies on fluorinated liquids include the liquid-state structure of hydrogen fluoride [11], fluorosulfuric acid [12], hexafluoro-iso-propanol (HFIP)–water mixtures [13–16] and halogeno–ethanol–water mixtures [17]. The latter systems also employed X-ray and neutron diffraction with Empirical Potential Structure Refinement (EPSR) modelling that showed microhetrogeneities on the length scale of a few nanometers due to clustering. HFIP–water mixtures for example are associated with a change from a tetrahedral-like water network structure to a chain-like water structure at high HFIP molecule concentrations. More broadly, the majority of diffraction, spectroscopic and modelling studies on aqueous solutions have focused on binary alcohol-water mixtures [18–20], usually at high concentrations (greater than 10 mole%) to maximize the signal between the solute and water molecules. Of note, neutron diffraction experiments have indicated that in high-concentration methanol–water mixtures, evidence for separate percolating networks coexists, despite both liquids being fully miscible in all proportions [19]. Tang *et al*. [18] have suggested that nanoscale segregation of water near molecules containing both hydrophobic and hydrophilic groups is commonplace and can lead to anomalous properties in heat capacity, vapour pressure and acidity. In this paper, we apply modern high-energy X-ray small- and wide-angle diffraction techniques combined with EPSR modelling to elucidate the structure of neat fluoroalkyl acids and acids in aqueous solutions to investigate these molecular interactions and their aggregation over a wide range of lengthscales.

## 2. High-energy X-ray diffraction

**Figure 2.**
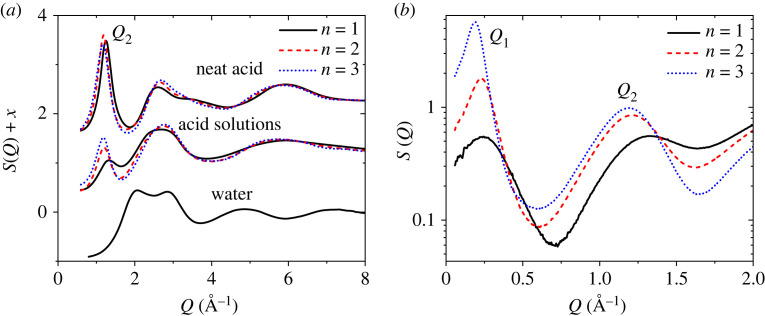
(*a*) The WAXS structure factors of neat acids CF_3_[CF_2_]*_n_*COOH where, *n* = 1, 2 and 3, diluted water 10 : 1 acid solutions and pure water. (*b*) The measured SAXS structure factors for the 10 water : 1 acid solutions on a log *y*-scale. *Q*_1_ and *Q*_2_ denote the first two peaks in the SAXS signal.

The X-ray pair distribution function (PDF) method is a well-established technique for the characterization of both local and intermediate range ordering of disordered organic materials, providing details of molecular structure at the atomic level [21]. Pure perfluoroalkyl carboxylic acid chains of CF_3_[CF_2_]*_n_*COOH, where *n* = 1, 2, 3 (figure 1), and their aqueous solutions of 1 acid : 10 water molecules were loaded into 2 mm diameter, thin-walled (0.1 mm) capillaries, and measured at 25°C. The high-energy X-ray small-angle and wide-angle X-ray scattering (SAXS/WAXS) measurements were performed on beamline 6-ID-D at the Advanced Photon Source at Argonne National Laboratory. The set-up and correction procedures have been previously described in detail [22], whereby the SAXS data were normalized to the WAXS data over a substantial overlap region, which for high-energy X-ray diffraction means essentially normalizing to the number of electrons in the system at high-*Q*. Experiments were carried out using a monochromatic X-ray beam *E* = 100 keV (*λ* = 0.124 Å) collimated to a square 0.5 mm cross-section, and the scattered beam measured using a Varex CT4343 area detector. A NIST CeO_2_ powder standard was used for the WAXS sample-detector distance calibration, which was set to 360 mm in order to balance resolution and *Q*-range, where 0.6 < *Q*(Å^−1^) < 25.7. A silver behenate powder standard was used for the SAXS sample-distance calibration, corresponding to 1430 mm, where 0.05 < *Q*(Å) < 3.3. The SAXS and WAXS data were analysed as described previously [23] using the software *Fit2D* [24] and *PDFgetX2* [25] and combined to yield S(*Q*) over the entire *Q*-range, figure 2. In brief, dark current, geometrical effects, beam polarization, background and attenuation corrections were applied to all datasets to yield the total X-ray structure factors S(*Q*) and differential PDFs D(*r*) [22].

## 3. Empirical Potential Structure Refinement modelling

To investigate the hydrogen bonding and hydrophobic behaviour of the neat short-chain fluorinated acids and aqueous solutions, EPSR modelling was used [26] to obtain atomistic models based on the high-energy X-ray diffraction data. The EPSR simulations were performed on 150–230 acid molecules for the neat liquids (to maintain a cubic box length of approx. 30 Å) using atomic number densities of 0.075 atoms Å^−3^, in order to assess the validity of the method. Larger models containing 300 acid molecules plus 3000 waters (dilute solutions) within a cubic box of length approximately 50 Å were assembled using atomic number densities of 0.09 atoms Å^−3^ for the solutions to capture the observed SAXS features. EPSR is essentially a rigid body simulation but molecular flexibility can also be introduced, so here rotations of bonds along the fluoroalkyl chain and carboxyl group were enabled to provide a better fit. The parameters for the Lennard-Jones parameters and effective charges are illustrated in figure 1 and the electronic supplementary material, information.

**Figure 3.**
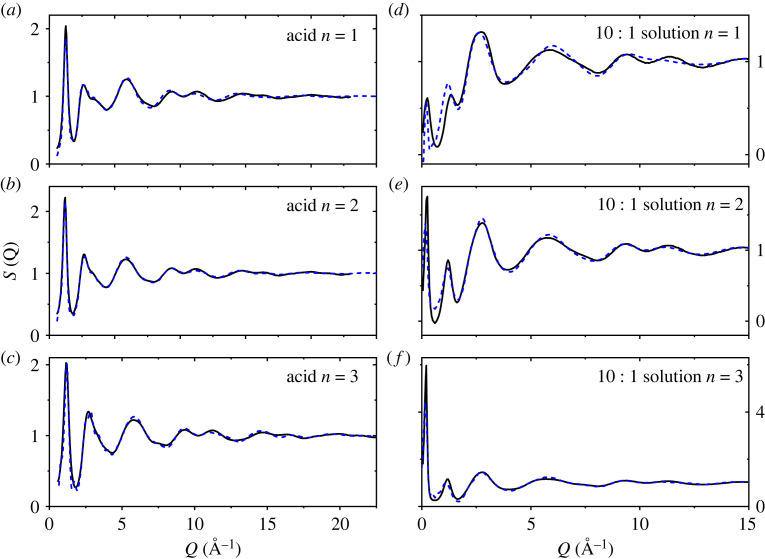
Measured X-ray S(*Q*)'s (black solid lines) and EPSR fits (dashed blue lines) to that of (*a–c*) neat liquid CF_3_[CF_2_]*_n_*COOH where, *n* = 1, 2 and 3, and (*d–f*) diluted water 10 : 1 acid solutions for *n* = 1, 2 and 3.

Following the initial Monte Carlo equilibration, the empirical potential term was refined to improve agreement with scattering data. Once the goodness-of-fit parameter was minimized between the model and the experimental S(*Q*), structural data were collected over ensembles of at least 2000 configurations, figure 3. Overall, good fits were found for the neat acids; however, finding a balance for fitting both the SAXS and WAXS regions of the solutions simultaneously proved more problematic, particularly for the *n* = 1 solution, although reasonable compromises were eventually found. While the EPSR fit to the data does not necessarily give a unique structural three-dimensional configuration of molecules, it does provide important insight into the types of interactions that are likely in the liquid state. Since X-rays are scattered by electrons, the S(*Q*)s and corresponding PDFs are most sensitive to the heavier carbon, oxygen and fluorine atoms, i.e. the chain backbones and centres of the water molecules.

## 4. Results

As shown in figure 2, the measured low-*Q* SAXS peak position in the solution spectra decreases systematically with increasing acid chain length, from *Q*_1 _= 0.245 ± 0.005 Å^−1^ (for *n* = 1), to 0.23 Å^−1^ (*n* = 2) and 0.19 Å^−1^ (*n* = 3). The intense *Q*_1_ peak for *n* = 3 dominates the X-ray structure factor and is ×10.5 larger than the peak in *n* = 1 and ×3.2 larger than the peak in *n* = 2. The sharpness of the *Q*_1_ peaks indicates the presence of well-defined periodicities in real space that extend beyond the size of our cubic simulation box, i.e. greater than 50 Å length. However, our attempts to scale up to 100 Å box length resulted in unreasonable equilibration times. Similar issues have been reported on Monte Carlo fits of other large-scale simulations [27]. The so-called first sharp diffraction peak (FSDP) in the WAXS signal, denoted here as *Q*_2_ in the solution S(*Q*)'s is known to be associated with intermediate range order in the liquid [21]. The FSDP peak positions are also found to decrease systematically with increasing acid chain length, from *Q*_2 _= 1.33 ± 0.01 Å^−1^ (for *n* = 1), to 1.21 Å^−1^ (*n* = 2) and 1.19 Å^−1^ (*n* = 3).

**Figure 4.**
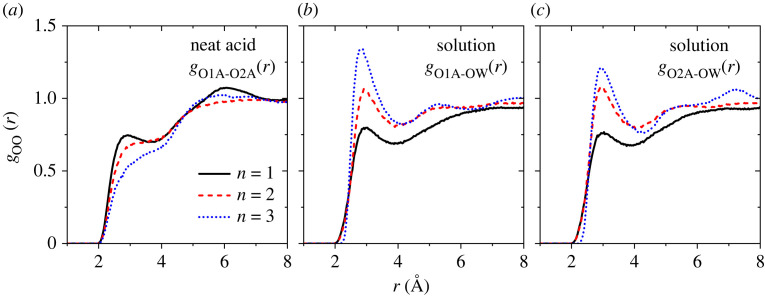
The EPSR oxygen–oxygen partial PDFs for (*a*) the acid hydroxyl oxygen–acid carbonyl oxygen *g*_O1A-O2A_(*r*) in the neat acids, (*b*) the acid hydroxyl oxygen–water oxygen *g*_O1A-OW_(r) in the solution mixture and (*c*) the acid carbonyl oxygen–water oxygen *g*_O2A-OW_(*r*) in the solution mixture.

Details of the molecular interactions between molecules in the pure acids and solutions are contained in the EPSR oxygen–oxygen partial pair correlations in figure 4. The neat acid molecular models show the liquid structure comprises clusters of winding hydrogen-bonded chains. The hydroxyl oxygen-carbonyl oxygen PDF *g*_O1A-O2A_(*r*) for the neat acid *n* = 1 is centred around *r*_OO _= 2.9 Å, and the corresponding coordination number is *n*_OO _= 1.0 integrating out to the first minimum at *r*_int _= 4.0 Å. As expected from steric hinderance considerations, the value of *n*_OO_ decreases with increasing acid chain length to 0.7 for *n* = 2 and 0.6 for *n* = 3, resulting in fewer hydrogen-bonded chains (because not every molecule is hydrogen-bonded). In the aqueous acid solutions, the hydrogen bonding between the acid molecules and water molecules increases significantly with increasing acid chain length. For *n* = 1, the carbonyl oxygen acceptor O2A shows the weakest interaction with water oxygen OW at a distance of *r*_OO _= 2.9 Å and has a coordination number of *n*_OO_ ∼ 3.2 (integrating out to *r*_int _= 4 Å). The value of *n*_OO_ increases to 3.8 for the *n* = 2 solution and 4.1 for *n* = 3. Concomitantly, the *n*_OO_ hydroxyl (donor) oxygen O1A–water oxygen coordination number also increases with increasing chain length, from 3.3 for *n* = 1, to 3.8 (*n* = 2) and 3.8 (*n* = 3). This indicates that the carboxylic ‘head’ on the smallest acid molecules hydrogen bonds less with the surrounding waters, most likely due to geometrical constraints. This is similar to the behaviour found in halogenoethanol-water mixtures, where the neat liquids are hydrogen-bonded to each other but separated due to steric hindrance, and as the water content increases a tetrahedral-like structure of water evolves as the halogenoethanol and water molecules cluster leading to heterogeneous mixing at the molecular level [17].

## 5. Discussion

**Figure 5.**
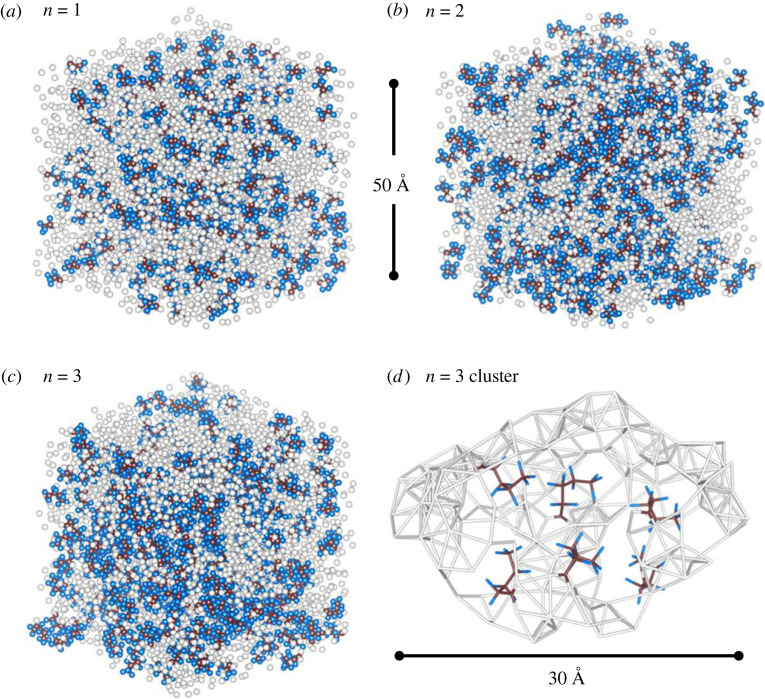
(*a–c*) The full three-dimensional EPSR simulation boxes for *n* = 1, 2 and 3 water 10 : 1 acid solutions, where water oxygens are plotted as white spheres and the acid molecules are brown (carbon) and blue (fluorine) for clarity. Hydrogen atoms have been omitted for clarity; (*d*) stick representation of a water cluster surrounding six *n* = 3 acid molecules, showing the regularity of the acid spacing and an adjacent dense water channel at the top.

Short-chain alcohols such as methanol, ethanol and propanol in aqueous solutions all form heterogeneous clusters at mole fractions greater than 10% [18,28]. Previous X-ray diffraction studies have indicated that hydrogen-bonded alcohol chains and tetrahedral-like water networks coexist at room temperature [28]. Furthermore, Li *et al*. [28,29] have suggested a structural transition occurs between isolated alcohol molecule concentrations that have independent hydration shells, and the inhomogeneous mixing of extensive hydrogen-bonded water networks and the aggregation of solute molecules at higher concentrations. Such nanoscale heterogeneity is highly composition dependent [18] since the maximum number of water molecules in the hydration shell surrounding a single-alcohol molecule increases with the length of the carbon chain of the alcohol [29]. Snapshots of our three-dimensional EPSR simulation boxes for the short-chain perfluoroalkyl carboxylic acid solutions also reveal the nanoscale aggregation of water molecules, figure 5*a–c*. With increasing acid chain length, the models show a systematic increase in the acid–water O_A_–O_W_ correlations (with similar magnitudes for the acid donor–water acceptor and acid acceptor–water donor hydrogen-bonding interactions). Most of the acid–water interactions involve hydroxyl and/or carboxyl groups hydrogen bonding to water molecules in densely packed channels, although many other interactions in close proximity are non-bonded O_A_–O_W_ interactions.

### (a) Nanoscale aggregation

Inspection of the EPSR *Q*-space models reveals that the *Q*_2_ peak arises mainly from S_C-C_(*Q*) (carbon–carbon) interactions, i.e. acid–acid correlations originating from the molecular backbones. It can be seen in figure 5*d* that adjacent (non-bonded) acid molecules are relatively evenly distributed throughout the simulation box giving rise to the periodicity associated with *Q*_2_. The *Q*_1_ peak in the SAXS signal is more complex and mostly originates from both the S_C-C_(*Q*) and S_OW-OW_(*Q*) (water–water) partial structure factors with a periodicity of 2π/(*Q*_1_) ∼ 30 Å. This corresponds to density fluctuations from both acid–acid and water–water correlations, respectively, due to clustering, that become more strongly correlated as the acid molecule size increases. The peak in S_C-C_(*Q*) is attributed to groups of acid molecules and the peak in S_OW-OW_(*Q*) from the aggregation of water molecules surrounding these clusters.

**Figure 6.**
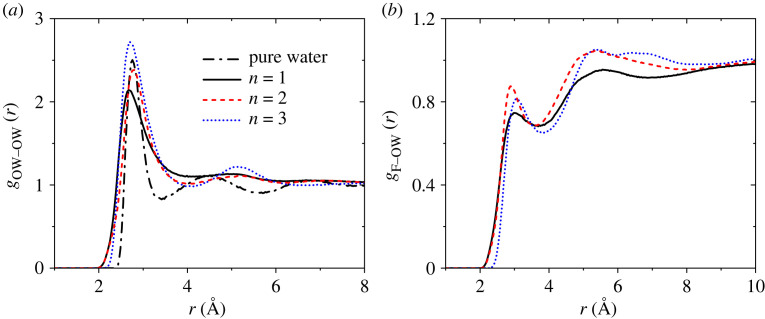
The EPSR partial PDFs for (*a*) water oxygen–water oxygen O_w_–O_w_ and (*b*) the acid chain fluorine–oxygen water F–O_w_.

Next, we investigate the changing network topology with acid chain length. Figure 6*a* shows that the water network, denoted by the O_W_-O_W_ partial PDF is significantly broadened compared with pure liquid water, indicating a highly disordered network containing several non-bonded correlations. The O_W_-O_W_ coordination number in an open tetrahedral-like pure water is 4.3 (integrating out to *r*_int _= 3.3 Å [30]). This compares with lower *n*_OWOW_ values of 3.7 (for *n* = 1), 3.6 (*n* = 2) and 3.8 (*n* = 3) in the acid solutions, with typical errors of ±0.3 over the same range. Integrating out to the minima in *g*_OWOW_(*r*) at *r*_int _= 4 Å the coordination number *n*_OWOW_ increases to approximately 6.4 water molecules, with 4.4 ± 0.5 bonds/molecule if the constraint of an intermediate hydrogen bond is imposed, i.e. on average there are approximately two non-bonded water molecules within the first coordination shell. In addition, the intensity of the first (approx. 2.7 Å) and second (at approx. 5.2 Å) shells in *g*_OWOW_(*r*) for the acid solutions increases with longer chain lengths, which can be associated with an increase in the local ordering of water molecules. Overall this indicates the presence of densely packed water channels containing a mixture of bent hydrogen bonds and non-bonded molecular interactions.

### (b) Solvation shells

By contrast, the hydrophobic-water partial PDF *g*_F-OW_(*r*) in the solutions shows the first peak around 3.0 Å and the corresponding *n*_FO-W_ coordination numbers decrease slightly from 1.8 (*n* = 1), 1.8 (*n* = 2) to 1.5 (*n* = 3, integrating out to *r*_int _= 3.5 Å). That is to say, each F atom on the chain is surrounded by approximately two water molecules on average, figure 6*b*. The first and second (5.4 Å) nearest neighbour F-O_W_ peaks in *g*_F-OW_(*r*) increase in intensity from *n* = 1 to *n* = 2. For the longest chain length (*n* = 3 solution), there is a slightly stronger repulsion as the first peak distance increases by approximately 0.1 Å, and there is a loss of intensity in the 4.3 Å region between the first and second nearest neighbour shells.

**Figure 7.**
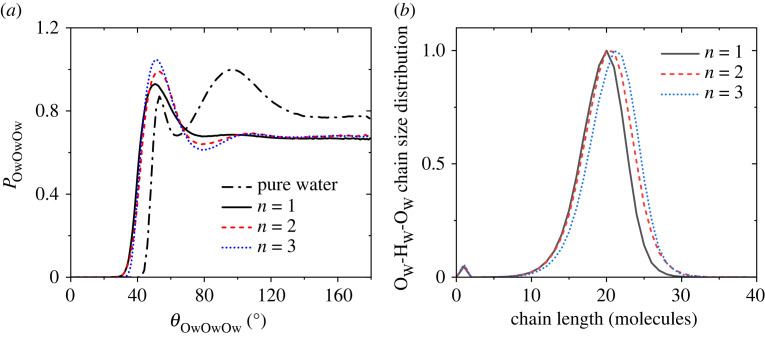
(*a*) The normalized -O_W_-O_W_-O_W_- bond-angle distribution for water molecules in the *n* = 1, 2 and 3 acid solutions compared with pure water. (*b*) The -O_W_-H_W_-O_W_- chain size distributions from the EPSR models in the water 10 : 1 acid solutions for *n* = 1, 2 and 3. Obtained using cut-offs of *r*_O-H _= 2.4 Å and *r*_O-O _= 4.0 Å.

The nanoscale heterogeneity leads to the question of how much the structure of hydrogen-bonded water is affected by the presence of short-chain perfluoroalkyl carboxylic acids? The -O_W_-O_W_-O_W_- bond-angle distributions for water molecules in the *n* = 1, 2 and 3 acid solutions in figure 7*a* show the water molecule arrangement to be notably different from the tetrahedral-like distribution found in pure water. For pure water at room temperature, the -O_W_-O_W_-O_W_- bond-angle distribution has a main tetrahedral peak at 96° (close to the perfectly tetrahedral peak at 109.5°) and a smaller non-bonded interstitial peak at 53° [31]. By contrast, the -O_W_-O_W_-O_W_- bond-angle distributions for acid solutions have the main peak around 52° and only a very small broad peak persists at approximately 108°. These bond-angle distributions sharpen with increasing chain length. The presence of short-chain perfluoroalkyl carboxylic acids therefore causes a major perturbation to the water structure as solvation shells are formed by chains of water molecules with bent hydrogen bonds due to the restricted geometrical arrangements.

To investigate the distorted hydrogen-bonded water arrangement further, we calculated the -O_W_-H_W_-O_W_- chain size distributions from the EPSR models in the water 10 : 1 acid solutions for *n* = 1, 2 and 3 (using cut-offs of *r*_O-H _= 2.4 Å and *r*_O-O _= 4.0 Å). The chain lengths were calculated using the shortest path criterion, i.e. the path with the least number of linkages between two molecules. All acid solutions show a primary chain size distribution centred on approximately 20 molecules that shifts slightly to larger chain lengths for longer acid chain molecules, figure 7*b*. Only few (less than 1%) water molecules were found to be completely isolated (chain length = 1).

## 6. Conclusion

Small-and-wide angle X-ray scattering measurements on short-chain perfluoroalkyl carboxylic acids and solutions have been interpreted with EPSR modelling, which revealed distinct long-range interactions between both non-bonded chains of acid molecules and distorted hydrogen-bonded solvation shells formed by water molecules. This water structure is very different than the usually preferred tetrahedral-like network topology. The results reveal a rich variety of structures on varying length scales. Most notably, in tandem with the regularity between non-bonded acid molecules in the liquid, nanoscale heterogeneity was observed to occur in all three model solutions studied, and the nanoscale aggregation of molecules increased with increasing acid chain length. Within the solvation shells, carboxyl and hydroxyl groups on the short-chain perfluoroalkyl carboxylic acids preferentially hydrogen-bonded to adjacent dense water-rich regions. The presence of long-range structural ordering and aggregation effects in these molecular liquid mixtures is most likely highly composition- and size-dependent, so further study is necessary to better understand these relations in detail. It is anticipated that these preliminary results will aid in the benchmarking and development of realistic interatomic potentials, so that accurate computer simulations can be performed at more dilute concentrations relevant to current environmental challenges.

## Data Availability

The datasets supporting this article have been uploaded as part of the electronic supplementary material [32].
